# Draft Genome Sequence of *Geobacillus thermoleovorans* Strain B23

**DOI:** 10.1128/genomeA.00944-13

**Published:** 2013-11-14

**Authors:** Chanita Boonmak, Yasunori Takahasi, Masaaki Morikawa

**Affiliations:** Division of Biosphere Science, Graduate School of Environmental Science, Hokkaido University, Sapporo, Japan

## Abstract

Here, we report the draft genome sequence of *Geobacillus thermoleovorans* strain B23, which was isolated from a deep subterranean petroleum reservoir in Japan. An array of genes related to unique long-chain alkane degradation pathways in *G. thermoleovorans* B23 has been identified by whole-genome analyses of this strain.

## GENOME ANNOUNCEMENT

An extremely thermophilic bacterium, *Geobacillus thermoleovorans* strain B23, was previously isolated from production water at a 2,150-meter depth (105°C) of a subterranean petroleum reservoir in Niigata, Japan (1). B23 can degrade a broad range of alkanes (C_11_ to C_32_) at 70°C by the terminal oxidation pathway. Despite extensive survey and analysis of related genes and enzymes in this pathway (2–4), the alkane monooxygenase system for initial oxidation of alkane to primary alcohol has remained unknown. In order to elucidate comprehensive alkane degradation pathways in *G. thermoleovorans*, whole-genome sequence analysis was conducted.

Genomic DNA from *G. thermoleovorans* B23 was prepared using the Marmur method (5). Paired-end libraries of 8-kb (±20%) fragments were generated from genomic DNA and sequenced using the Roche 454 GS FLX (Basel, Switzerland) 1.4 region at the Hokkaido System Science Co., Ltd. (Sapporo, Japan), achieving approximately 25× average genome coverage. The genome sequencing generated 210,521 raw reads covering a total of 87,444,156 bp. There were a total of 209 contigs of >100 bp, with a total of 3,353,053 bp, and 150 large contigs of >500 bp, with a total of 3,337,458 bp, generated using GS De Novo Assembler v.2.8. The Q40 plus bases showed 99.94% reliability. The total G+C content was 52.3%. The assembled B23 draft genome sequence was annotated by using the xBASE bacterial genome annotation service (http://www.xbase.ac.uk/annotation/) and MiGAP microbial genome annotation pipeline (http://www.migap.org). There were 3,349 protein-coding sequences (CDS), 10 rRNA genes, and 84 tRNA genes. The sequence reads were also aligned with the *Geobacillus kaustophilus* HTA426 (6) reference genome using the multiple-genome alignment program Mauve (7). An in-depth comparative genomic analysis of these data will be included in a future publication.

### Nucleotide sequence accession numbers.

The draft genome sequence of *G. thermoleovorans* B23 is available in DDBJ/EMBL/GenBank under the accession numbers BATY01000001 through BATY01000209.
